# Diagnosis of the Primary Tumor Site in the Case of Liver Metastatic Carcinoma

**DOI:** 10.7759/cureus.26782

**Published:** 2022-07-12

**Authors:** Mercedes Malone, Christopher Bray, Xiaolong Sean Liu

**Affiliations:** 1 Internal Medicine, Hospital Corporation of America (HCA) Florida North Florida Hospital, Gainesville, USA; 2 Internal Medicine, University of Central Florida College of Medicine, Graduate Medical Education/Hospital Corporation of America (HCA) Florida North Florida Hospital, Gainesville, USA; 3 Pathology, Hospital Corporation of America (HCA) Florida North Florida Hospital, Gainesville, USA

**Keywords:** hepatic malignancy, lung adenocarcinoma, non-cirrhotic liver, metastatic adenocarcinoma of unknown primary, immunohistochemistry staining

## Abstract

Immunohistochemical markers have been frequently utilized as a diagnostic tool in pathology to help in diagnosing malignancy of unknown primary sites. In previous cases, the immunohistological expression of cytokeratin 7 (CK7), Napsin A, and thyroid transcription factor-1 (TTF-1) has helped identify and diagnose primary malignancy as originating from the lung. This case report describes an elderly woman with a liver metastasis consistent with a lung primary and illustrates the utility and importance of tissue-specific markers as a diagnostic tool in the evaluation of unknown primary tumors.

## Introduction

Carcinoma of unknown primary origin is one of the most prevalent malignancies. Patients in whom a site of origin can be determined have superior outcomes compared to those in which the primary site remains unidentified [1]. The differentiation between metastatic carcinoma to the liver and primary hepatocellular carcinoma may be particularly challenging. Approaches to diagnosing and determining the primary origin of carcinoma include clinical and radiological evaluations, biopsy, morphological evaluation, and, increasingly, immunohistochemistry [2]. The presented case highlights the critical importance of immunohistochemical markers in identifying the primary sites of malignancy for earlier intervention.

## Case presentation

An 82-year-old female with a past medical history of hypertension, prediabetes, reactive airway disease, anxiety, and glaucoma with recent hospital admission for a high-grade pyloric obstruction treated with a gastrojejunostomy tube and a recurrent, complex liver abscess treated most recently with drainage and antibiotics presented to our hospital emergency department (ED) with a chief complaint of intractable nausea and vomiting. The patient has had a history of tobacco use disorder for several years. She had a surgical history of urological treatment of recurrent, high-grade ureteropelvic junction obstruction and hydronephrosis treated with stenting and pyeloplasty, occurring between seven and 12 years prior to admission. Her family history was unremarkable. A month prior to her admission to our hospital, she had completed a one-month course of intravenous antibiotics for a complex liver abscess that was found on CT imaging. She had been experiencing multiple episodes of intractable non-bloody and non-bilious vomiting associated with poor oral intake due to nausea. Upon admission to the hospital, she was afebrile, and her vital signs were within normal limits. A physical examination was significant for mild abdominal distention without any guarding, ascites, or pain upon palpation.

Routine laboratory studies were obtained (Table 1). The complete blood count was significant for leukocytosis and thrombocytosis, judged as likely reactive in nature. The patient had slight hypokalemia of 2.9 mmol/L, likely due to multiple episodes of emesis. Her liver function enzymes were within normal limits.

**Table 1:** Pertinent laboratory data upon admission

Laboratory parameters	Levels upon admission	Normal ranges
White blood cell count	25.3×10^3^/uL	4-10.5×10^3^/uL
Hemoglobin	12.8 g/dL	11.2-15.7 g/dL
Hematocrit	41.3%	34.1%-44.9%
Platelet count	465×10^3^/uL	150-400×10^3^/uL
Sodium	136 mmol/L	136-145 mmol/L
Potassium	2.9 mmol/L	3.5-5.1 mmol/L
Creatinine	0.87 mg/dL	0.60-1.30 mg/dL
Glucose	191 mg/dL	74-106 mg/dL
Lactic acid	1.3 mmol/L	0.4-2 mmol/L
Total bilirubin	0.3 mg/dL	0.2-1 mg/dL
Aspartate aminotransferase	36 units/L	13-56 units/L
Alanine aminotransferase	36 units/L	13-56 units/L
Alkaline phosphatase	68 units/L	45-117 units/L
Carcinoembryonic antigen	85.6 ng/mL	0-5 ng/mL
Alpha-fetoprotein	1.8 ng/mL	0-8.3 ng/mL
CA 19-9 antigen	457 units/mL	0-30.9 units/mL
CA 125 antigen	<20 units/mL	0-30.2 units/mL

**Figure 1:**
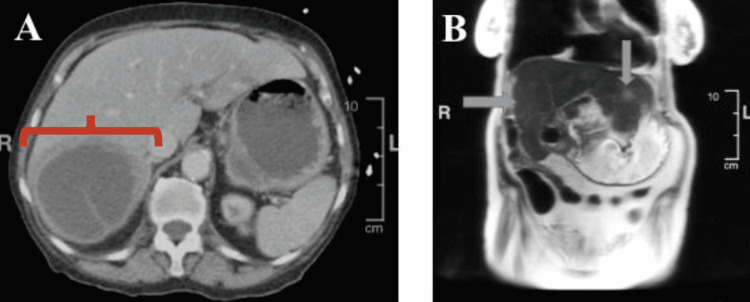
CT images of abdominal complex liver mass and MRI illustrating hepatic malignancy A: Noncontrast CT scan demonstrating a complex cyst in the right lobe liver extending up to the dome of the liver and measuring approximately 10 cm in diameter. There is a second fluid collection inferiorly in the right lobe liver that likely represents the gallbladder. B: MRI of the abdomen illustrating areas of abnormal enhancement in the liver concerning for metastasis.

A CT scan with contrast of the abdomen and pelvis illustrated a complex cystic liver mass that was relatively unchanged from a prior image taken before the recent completion of a course of antibiotics (Figure 1A). Because of suspected malignancy, her alpha-fetoprotein, carcinoembryonic antigen (CEA), CA 125 antigen, and CA 19-9 antigen were all tested. Both her CEA and CA 19-9 were elevated beyond the normal reference range. While CEA is often found elevated and utilized during treatment for adenocarcinomas of the gastrointestinal system, it can also be elevated in breast, lung, ovarian, and pancreatic cancer and in non-malignant conditions such as smoking, alcoholism, and pancreatitis [3]. While CA 19-9 is often found elevated and utilized during treatment for pancreatic carcinomas, it can also be elevated in lung, gastric, biliary tract, and colorectal cancer [3].

The patient underwent a CT-guided placement of a 12-French drain inserted into the complex liver cyst. Pus was aspirated from the liver cyst, and samples were sent for culture and cytology. The patient experienced some relief of her symptoms but continued to have a distended abdomen and subsequently underwent an MRI of the abdomen with contrast that identified a large mass in the right lobe of the liver that appeared significantly smaller in size status post drainage, a finding consistent with an abscess. However, the MRI also illustrated an additional complex lesion in the region of the gallbladder fossa with abnormal enhancing tissue extending into the surrounding lung parenchyma as well as adjacent soft tissue extending outside the liver into the adjacent distal stomach (Figure 1B). The intrahepatic duct in the posterior right lobe was also dilated due to an obstruction from metastasis. There were also mildly enlarged and enhancing right retrocrural lymph nodes suggesting potential metastasis as well. The MRI findings were considered highly suggestive of malignancy and were particularly concerning for metastasis given the presence of other foci of abnormal enhancement throughout the liver.

**Figure 2:**
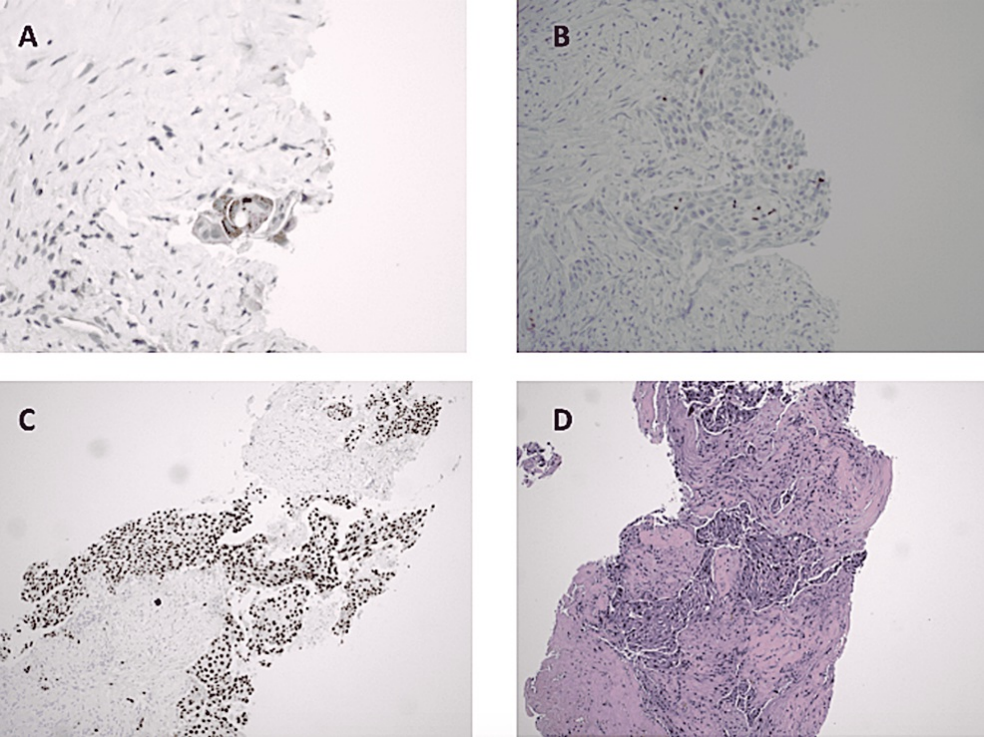
Histopathology: H&E and immunohistochemistry of liver mass on needle core sections A: Napsin A: similar to TTF-1, a few tumor cells are positive for Napsin A. B: TTF-1: a few tumor cells form neoplastic glands and are positive for TTF-1. C: p40: neoplastic squamous cells are highlighted. D: H&E staining of liver biopsy: tumor shows both squamous cell differentiation and glandular formation.

The patient underwent a percutaneous needle core biopsy of segment 4 of the liver. The specimen (needle core biopsy) was submitted for pathology evaluation. The final pathologic diagnosis was based on the microscopic examination of the sections prepared from the submitted tissue (Figure 2), and it showed multiple tumor nests in the desmoplastic stroma with squamous differentiation and several small foci with features suggestive of glandular formation (Figure 2D). By paraffin section immunohistochemistry, the tumor stained positive for p40, cytokeratin 7 (CK7), thyroid transcription factor-1 (TTF-1), and Napsin A (Table 2) and negative for CK20, CDX2, AFP, GLY-3, HepPar-1, and PAX-8 (data not shown). The morphology and immunoprofile were most consistent with adenosquamous carcinoma of lung primary origin [4]. The patient also had a duodenum biopsy that was significant for mild peptic inflammatory changes without any sign of malignancy.

**Table 2:** Liver mass: morphologic evaluation and paraffin section immunohistochemistry Upon microscopic examination, both squamous cell differentiation and occasional glandular formation were identified. The positive p40 and GATA binding protein 3 (GATA-3) are in keeping with squamous cell differentiation, and the positive TTF-1 and Napsin A are in keeping with a glandular component of lung primary in the tumor. In this setting, metastatic adenosquamous carcinoma of lung primary origin is favored.

Positive	Negative
p40	CK20
CK7	CDX2
TTF-1 (focal, weak)	AFP
Napsin A (focal, weak)	GLY-3
GATA-3 (weak)	HepPar-1
ER (weak)	PAX-8

**Figure 3:**
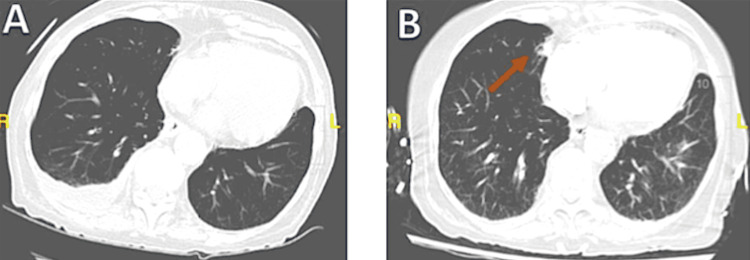
CT images of the lung without and with suspicion area possible for malignancy A: Noncontrast CT scan that was taken two months prior did not have any abnormality present. B: CT scan of the lung two months later from Figure 3A illustrates an area of abnormality that may possibly be a malignancy that had not been present previously (arrow). CT: computed tomography

Ultimately, the patient was intubated due to respiratory decline. Hospice care was pursued, and the patient passed away due to septic shock likely from bowel ischemia. Shortly after the patient’s death, the patient’s pulmonary CT scan was reviewed by radiology because of the suspected diagnosis of an adenosquamous lung carcinoma suggested by the pathology. A comparison of the noncontrast CT scan to another CT scan of the lung performed two months later revealed a suspicious area that might have been considered a possible malignancy that was previously not present (Figure 3A, 3B).

Post-mortem, the patient’s outpatient records (for the past 15 years) were received, and it was found that the patient had an even more extensive smoking history than reported on admission, which likely predisposed her to this probable lung carcinoma. The patient also had a screening colonoscopy seven years prior to her admission to our hospital with no sign of underlying colorectal malignancy. Other cancer screenings including gynecologic and breast cancer screening were unknown as she had been declining further screening.

## Discussion

When a liver mass is identified in a patient with a non-cirrhotic liver, the chances of the mass being a metastasis from an alternative site is significantly greater than it being a hepatic primary tumor. One study suggests that the chances of a hepatic malignancy in a non-cirrhotic liver as metastatic in nature may be as high as 86% relative to hepatic primary [5].

It is imperative for physicians to identify the primary site of a metastatic malignancy as early as possible as this will guide critical, often tumor-specific, treatment and management going forward. There is significant harm associated with missing the site of a primary tumor, especially in light of the many tumor-specific treatments that now exist to target specific malignancies. In the present case, the patient had multiple liver masses that were initially presumed to be pancreatobiliary in origin. However, due to pathology evaluation, particularly with relatively lung-specific positive tumor markers by immunostains, an alternative primary site was suggested. This was supported by comparing imaging studies.

The core needle biopsy of the liver mass illustrated immunoreactivity for the following tumor immunohistochemical markers in our patient: TTF-1, CK7, Napsin A, and p40. There were no molecular studies performed. TTF-1, CK7, and Napsin A collectively are highly diagnostic of lung adenocarcinoma and p40 typically for squamous lung carcinoma [6]. The results pointed toward a non-small cell lung carcinoma. Approximately 10%-14% of hepatic metastasis is believed to originate from non-small adenocarcinoma [7].

Although the initial reading of the radiological images of the patient’s lung did not illustrate any obvious or visible nodule or mass, a postmortem review of pulmonary images illustrated an area that was not present before and is suspicious of malignancy. This, in combination with her extensive smoking history and the other unremarkable screening, make adenosquamous carcinoma of the lung the more likely diagnosis. A PET-CT or lung biopsy would be required to enhance the certainty of this diagnosis and optimize therapeutic options, but the patient unfortunately opted for hospice care prior to these diagnostics. A pancreatobiliary malignancy, especially in the setting of the pyloric obstruction, was a consideration, but the pathology combined with the new spiculated lung mass makes this less likely. While an endoscopic retrograde cholangiopancreatography (ERCP) or endoscopic ultrasound may have provided additional support, this was not feasible given her condition.

## Conclusions

This case illustrates that immunohistochemical markers are critical in identifying primary sites of malignancy and can lead to earlier treatment and decreased cost of management. There are instances where it is not easy to identify the primary site of malignancy. Even a small lung malignancy can lead to distant liver metastasis. The site of the primary is oftentimes missed upon radiological examination due to its resemblance to other pulmonary artifacts such as atelectasis. Immunohistochemistry stains such as TTF-1, CK7, Napsin A, and p40 play a significant role in the process of identifying the primary origin of malignancy and are a valuable tool in diagnosing and identifying the original site of a liver tumor.
